# Use of Sublingual Immunotherapy for Aeroallergens in Children with Asthma

**DOI:** 10.3390/jcm9103381

**Published:** 2020-10-21

**Authors:** Carlo Caffarelli, Carla Mastrorilli, Michela Procaccianti, Angelica Santoro

**Affiliations:** 1 Clinica Pediatrica, Dipartimento di Medicina e Chirurgia, Università di Parma, Azienda Ospedaliero-Universitaria di Parma, 43126 Parma, Italy; michela.procaccianti@outlook.it (M.P.); angelica.santoro204@gmail.com (A.S.); 2 UO Pediatria e Pronto Soccorso, Azienda Ospedaliero-Universitaria Consorziale Policlinico, Ospedale Pediatrico Giovanni XXIII, 70126 Bari, Italy; carla.mastrorilli@icloud.com

**Keywords:** asthma, children, efficacy, house dust mite, pollen allergy, rhinitis, safety, sublingual immunotherapy

## Abstract

Asthma is a heterogeneous disease that in children is often allergen-driven with a type 2 inflammation. Sublingual immunotherapy represents an important progress in the use of personalized medicine in children with allergic asthma. It is a viable option for house dust mite-driven asthma and in subjects with the asthma associated with allergic rhinitis. The use and indications for isolated asthma caused by other allergens are still controversial owing to heterogeneity of commercially available products and methodological limitations of studies in children. Nevertheless, most studies and meta-analyses found the efficacy of sublingual immunotherapy. Sublingual immunotherapy is safe but cannot be recommended in children with uncontrolled asthma.

## 1. Introduction

Asthma is a chronic disease affecting 10%–15% of school-aged children [1,2]. First-line treatment for allergic asthma consists of such medicines as inhaled corticosteroids, long-acting beta2-agonists and short-acting beta2-agonists as needed with the aim of minimizing symptoms, improving lung function, and reducing inflammation. Notwithstanding, some children need additional treatment to improve asthma control. Moreover, in patients who achieve control, symptoms can return upon discontinuation of drugs. Different response to the same therapy can be due to a distinctive asthma endotype that indicates a subtype of the disease with a distinct underlying pathophysiological mechanism. In childhood, two forms of asthma have been conventionally studied. The type2 (T2)-high endotype can be allergic [3] or nonallergic and it is characterized by an eosinophilic airway inflammation, while in the T2-low endotype, a neutrophilic or paucigranulocytic airway inflammation is found [4]. Most children have allergic asthma that may be considered a phenotype, with early onset, atopic background, family atopic history, allergic sensitization to common inhaled allergens, eosinophil inflammation, and bronchial hyperreactivity that overlaps with eosinophilic and T2 asthma [5]. In this promising era of precision medicine, matching asthmatic patients with the T2-high endotype allows “personalizing” more effective therapeutic choices that target the airway T2 pathway. They include biologicals and allergen specific immunotherapy (AIT) in asthmatics who partly respond or do not respond to first-line treatment or have a recurrence after a suspension. AIT has been the first attempt of precision medicine and it is tailored to the specific IgE that elicits the reaction. AIT is the only disease-modifying treatment for patients with IgE-mediated allergy due to airborne allergens. It consists of repetitive administration of the allergen extract that provokes symptoms with the purpose of inducing allergen tolerance in allergic asthma by targeting the underlying mechanisms and modifying the immunological response. Subcutaneous AIT (SCIT) has been the only accepted effective AIT for allergic rhinitis and asthma over several years [6] and it still represents the standard treatment for hymenoptera venom hypersensitivity. SCIT may rarely induce unpredictable anaphylactic reactions. Moreover, children can be annoyed by repeated injections that require visiting a doctor’s office. So, alternative safer and more comfortable routes of allergen administration that may allow self-administration at home have been investigated. The first randomized double-blind placebo-controlled trials (RDBPCT) of such routes took place in 1986 and studied sublingual AIT (SLIT) [7]. Subsequently, a remarkable number of clinical studies on SLIT was published showing indirectly an efficacy not far from that of SCIT [8,9] even if head-to-head studies are lacking. SLIT has been quickly recognized in official documents as an alternative to SCIT in respiratory allergy at variance from other routes [10,11,12,13,14]. Furthermore, SLIT has been used for other allergy-driven diseases, such as atopic dermatitis [15]. Both SCIT and SLIT share similar mechanisms, that involve induction of allergen-specific IgG4, stimulation of IgE-blocking IgG antibodies, T-cell tolerance [16]. These mechanisms suppress the specific Th2 immune response and prevent further exacerbations. In SLIT, an important role for antigen tolerance is played by the uptake of the allergen by dendritic cells of oral mucosa [17]. SLIT is specific for the allergen causing IgE-mediated asthma but not for asthma in itself [10]. So, we have analyzed the use of SLIT in asthmatic children, including an approach to its prescription that considers differences between allergens and suggestions for practice.

## 2. Product-Related Considerations

SLIT vaccines are available as liquid drops or tablets that are swallowed after keeping under the tongue for 1–2 min. Sublingual formulations are not equivalent since they vary according to the manufacturer in the diluent, preservatives, unit of measurement of potency, dosage, and schedules. The diversity in marketed products has led to heterogeneity in the way national regulators deal with different products. In most countries, AIT products usually require a marketing permission like other drugs [18]. However, SLIT products are also commercialized and routinely used in many countries as “named patient products” that just need to be prepared according to the Good Manufacturing Practice to be commercialized.

In the past 15 years, several big trials investigated orodispersible tablets with standardized determination of relevant allergen content of the extracts in RDBPCT involving a large number of children and adults with allergic rhinitis and/or asthma. Those studies characterized the optimal maintenance dose of each product [19]. Furthermore, they allowed for the approval of SLIT tablets for timothy, 5-grass, house dust mites (HDM), trees (birch), ragweed, and Japanese cedar by regulating authorities as medicinal products in several countries. Many SLIT products are marketed as solutions that are administered with a dropper, mini-pumps, or single-dose vials. The optimum dose with liquid extracts remains approximate [20] since trials evaluated a small number of children and they were not designed for registration. The mean number of children with asthma due to HDM in the active arm in 8 RDBPCTs [21,22,23,24,25,26,27,28] was 26 and the cumulative dose that was found to reduce asthma symptoms varied from 249.6 mcg Der P1 [23] to 1700 mcg Der P1 [26].

Regarding allergy to grass, SLIT with higher cumulative doses (4068 IR versus 18,031 IR) was associated with a significant low symptom/medication score [29].

SLIT with a mixed *Betula verrucosa*, *Corylus avellana* and *Alnus glutinosa* extract has a similar efficacy at a cumulative dose of 1.058 mcg (major pollen tree allergens) and of 8820 mcg (major pollen tree allergens) in 61 asthmatic children [30].

Cumulative effective doses for Par j varied from 20.3 mcg [23] to 52.2 mg [31].

At variance from SCIT, in SLIT, the build-up phase with increasing doses usually lasts a few days, or it is unnecessary, and the treatment starts with the maintenance dose. The maintenance dose can be administered according to the manufacturer: once a day, on alternate days, twice weekly [32]. SLIT for seasonal allergens can be discontinued at the beginning of the season (preseasonal treatment), at the end of the season (pre-coseasonal) or administered continuously. SLIT for perennial allergens is usually administered all year-round. A SLIT course of 3 years is recommended to achieve better long-term results [33,34,35]. However, a prospective study found that a treatment of 4 years slightly improved efficacy and long-term benefits in adults [36].

## 3. Sublingual Immunotherapy for Asthma

Several systematic reviews and trials have been conducted on the use of SLIT in asthmatic children. Meta-analyses have been hampered by heterogeneity among selected studies in population, allergens, products, outcomes, doses, duration of treatment. It is noteworthy that efficacy and safety should be characterized for each formulation because of differences between sublingual products. Furthermore, meta-analyses have been limited by power of the trials since most of them studied primarily patients with allergic rhinitis [16]. Allergic rhinitis, which affects 60–80% of asthmatic children [37,38], is the most frequent comorbidity and it is associated with worse asthma control. Furthermore, not validated instruments [1,39] were used and asthmatic exacerbations [40] at the time of the studies were not considered as the outcome that the authors should have tried to influence [2]. Even if these shortcomings questioned the conclusions [41], most meta-analyses and systematic reviews [6,42,43] showed the efficacy of SLIT in asthmatic children.

## 4. House Dust Mites

In asthmatic adolescents and adults, the findings of large studies [44,45,46] provided evidence of efficacy of HDM SLIT tablets. As a consequence, HDM SLIT has been incorporated in the Global Initiative for Asthma Report (GINA) recommendations [40] as an add-on treatment for HDM allergic asthma in adults with allergic rhinitis who have exacerbations despite a low-medium dose of inhaled corticosteroids if the forced expiratory volume per 1 s (FEV1) is greater than the 70% predicted. Therefore, patients with severe asthma receiving a high dose of inhaled corticosteroids [40] would not be given AIT. However, in the European Academy of Allergy and Clinical Immunology (EAACI) Guidelines [2], HDM SLIT tablets are recommended as an add-on treatment for adults with controlled and partially controlled HDM-driven allergic asthma irrespective of severity of asthma. In RDBPCT that included adolescents and adults, the efficacy of HDM SLIT tablets and drops has been shown [47,48] and they also have spared inhaled corticosteroid [49,50].

In children, ten RDBPTs [21,22,23,24,25,26,27,51,52,53] found that HDM SLIT drops improved asthma symptoms and reduced use of medication (Table 1). The systematic review by Rice et al. [39] found that HDM SLIT improved FEV1. An RDBPCT in a pediatric population reported negative results for HDM SLIT [28]. However, nearly all children had no asthmatic symptoms at the baseline so that lack of benefit could have been anticipated.

## 5. Grass Pollen

Several reports have shown the efficacy of grass pollen SLIT (Table 1). A large regulatory trial conducted with grass SLIT tablets in children with allergic rhinitis showed a significant improvement in the asthma symptom score but not in the medication use [54], while Rolinck-Werninghaus reported a decrease in the medication score [55]. Stelmach et al. [55] reported that grass SLIT significantly improved asthma symptoms and reduced the medication score in children. Dhami et al. [6] performed a systematic review and a meta-analysis of RDBPCTs on AIT for asthma in children and adults. They found that AIT was effective in decreasing the symptom score both in children and adults and the medication score in children and suggested (but not confirmed) in adults. AIT to grass pollen was effective in reducing the symptom score and the suggested (but not confirmed) medication score. Furthermore, SCIT was effective in reducing respiratory symptoms and drug consumption whereas SLIT was suggested but not confirmed to reduce the symptom and medication scores. However, only one study with SLIT in children [56] was reported [6]. If we look at real life, it has been reported that grass SLIT tablets for allergic rhinitis decrease the number of dispensed prescriptions for asthma medications [57].

## 6. Trees and Ragweed

Most data on AIT efficacy against tree pollen allergy have been shown in adult studies. A systematic review [42] reported two trials [55,58] showing the effectiveness of SLIT for asthma due to tree pollen allergy in children. A RDBPCT [59] found that SLIT to parietaria significantly reduced nonspecific bronchial hyperresponsiveness to methacholine. Recently, in a RDBPCT, Biedermann et al. [60] found that sublingual tablets containing a standardized birch extract were effective in 634 adolescents or adults with rhinoconjunctivitis caused by birch pollen and in the subpopulation with asthma and reduced the Asthma Control Test score.

Regarding ragweed, a RDBPCT by Nolte et al. [61] showed that ragweed SLIT tablets improved symptoms and medication use in children with rhinoconjunctivitis to ragweed pollen and reduced asthma symptoms and short-acting beta2-agonist use.

Data regarding efficacy of tree and ragweed SLIT in asthmatic children are reported in Table 1.

**Table 1:** Trials on SLIT efficacy carried out in children with asthma.

Author, Year	Trial	Allergen Extract	Age (Years)	Population (Active/Controls)	AIT Duration (Months)	Outcome
Tari et al., 1990 [21]	RDBPC	HDM	<12	30/28	30	↓ bronchial hyperreactivity
Hirsch et al., 1997 [22]	RDBPC	HDM	6–16	15/15	12	↓ asthma symptoms
Paino et al., 2000 [23]	RDBPC	HDM	8–15	12/12	24	↓ asthma symptoms ↓ medication use
Bahçeciler et al., 2001 [24]	RDBPC	HDM	8–15	7/8	6	↓asthma attacks ↑PEF
Ippoliti et al., 2003 [25]	RDBPC	HDM	5–12	47/39	6	↓asthma symptoms ↓ ECP, IL-13, PRL
Lue et al., 2006 [26]	RDBPC	HDM	6–12	10/10	6	↓asthma symptoms ↑ IgG4, total IgE ↓ eosinophil count ↑ lung function
Niu et al., 2006 [27]	RDBPC	HDM	6–12	49/48	6	↓asthma symptoms ↓ lung function
Pham-Thi et al., 2007 [28]	RDBPC	HDM	5–16	55/56	18	↓SPT reactivity ↑ lung function
Eifan et al., 2010 [51]	RCT	HDM	5–10	32/16	24	↓asthma symptoms ↓ medication score ↓ VAS ↓sIgE and SPT for HDM
Keles et al., 2011 [52]	RCT	HDM	5–12	48/12	18	↓asthma attacks, ↓ inhaled steroid dosage
Yukselen et al., 2012 [53]	RDBPC	HDM	6–14	21/10	12	↓ asthma symptoms ↓ medication use ↓ VAS ↓sIgE and SPT for HDM
Rolinck-Werninghaus et al., 2004 [55]	RDBPC	Grass	3–14	20/19	32	↓ medication score
Bufe et al., 2009 [54]	RDBPC	Grass	5–16	126/127	10	↓ asthma symptoms
Stelmach et al., 2009 [56]	RDBPC	Grass	6–17	25/25	24	↓ asthma symptoms ↑ FEV1 ↓ medication use
Vourdas et al., 1998 [58]	RDBPC	Olive	7–17	33/29	24	↓ asthma symptoms
La Rosa et al., 1999 [31]	RDBPC	Parietaria	6–14	20/21	24	↓ rhinitis symptoms ↓ SPT ↑ sIgG4
Pajno et al., 2004 [59]	RDBPC	Parietaria	8–14	15/15	24	↓ bronchial hyperreactivity
Valovirta et al., 2006 [30]	RDBPC	Tree pollen	5–15	59/29	17	↓ symptoms ↓ medication use
Nolte et al., 2020 [61]	RDBPC	Ragweed	5–17	513/512	7	↓ asthma symptoms ↓ short-acting beta2-agonist use

DBPC, double-blind placebo-controlled; EBC, exhaled breath condensate; ECP, eosinophil cationic protein; FEV1, forced expiratory volume in the 1st second; HDM, house dust mite; IL-13, interleukin 13; PEF, peak expiratory flow; PRL, prolactin; RCT, randomized controlled study; SPT, skin prick test; VAS, visual analog scale; ↑, increased; ↓, diminished.

## 7. Mold and Pet Allergens

The role of immunotherapy for allergens different from pollen and HDM is debated in asthma therapy. Mold allergies are frequent, especially in the Mediterranean area where 20% of allergic patients are sensitized [62,63]. Sensitization to molds is associated with a more severe progression of asthma [64]. AIT has been performed for *Alternaria* and *Cladosporium* but the use is limited by difficulty in obtaining a standardized allergen extract. Despite some evidence suggesting that specific AIT has a positive effect on respiratory symptoms, high quality studies are lacking. Several limitations characterize the available studies: many trials include both children and adults, small samples, absence of a placebo group. A meta-analysis [65] including nine randomized controlled studies (RCT) highlighted that low-strength evidence suggests that mold AIT is effective for respiratory symptoms. Just one of the selected studies was performed in children using *Alternaria* SCIT and it found an improvement of the symptom–medication score and the quality of life starting from the second year of administration. Regarding SLIT, in an RDBPCT [66], 27 patients aged 14–44 years with allergic rhinitis with or without intermittent asthma were treated with *Alternaria* SLIT. A significant reduction in symptoms, medication intake, and skin test reactivity in the active group was reported. So, the role of *Alternaria* immunotherapy in children remains unclear. There is little evidence for the use of SCIT but not of SLIT for *Cladosporium*.

To our knowledge, there is no study conducted exclusively on pediatric population regarding SLIT for animal dander [67]. An RDBPCT by Alvarez-Cuesta et al. [68] enrolling adolescents and adults with allergic rhinitis with or without asthma to cat dander showed an improvement of nasal symptoms but not of the bronchial symptom score, and a decreased PEF response to cat exposure in the SLIT group compared to the placebo group. Studies on SLIT for dander of other furry animals are lacking. Currently, high-quality studies on SCIT with dog allergen extracts have failed in asthmatic children [69].

## 8. The Effects of SLIT on Asthma Prevention

Allergic rhinitis predicts the development of asthma in children [70,71]. It has been documented that a three-year course of SCIT significantly reduced the occurrence of asthma in children with rhinitis caused by grass and/or birch pollen after 3 and 10 years [72]. Subsequently, two open RCT found that grass SLIT drops [73] and HDM, grass, birch SLIT drops [74] significantly reduced the risk of onset of asthma in children with allergic rhinitis after a course of 3 years. More recently, a large RDBPCT [75] has showed that grass SLIT tablets prevented respiratory symptoms and the use of asthma medication in children with allergic rhinitis. The results of the studies are reported in Table 2. In a large retrospective real-life study, Zielen [76] showed for the tablet formulation in patients >5 years of age that the relative risk reduction of asthma occurrence was around 30% during the treatment and around 40% during the follow-up. There is no evidence that AIT prevents development of new additional allergic sensitization in sensitized patients [77].

## 9. Safety

SLIT has been shown to be a safe treatment in many clinical trials and post-marketing surveys both in adults and in children, as well as in pre-school aged children [78], in children with allergic rhinitis or controlled asthma [79,80,81,82,83] (Table 3). SLIT has a better safety profile compared with SCIT and it can be safely given at home. Several RDBPCTs showed that the rate of systemic adverse events did not differ between the placebo and the active group [79]. Mild local adverse reactions are commonly reported [84]. They disappear within a few days of treatment. The well-known adverse events of SLIT mainly consist of oral itching or swelling, lip edema, throat pruritus, stomach ache. They are easily contained by transitorily diminishing the dose or antihistamine premedication for several weeks. Systemic reactions and asthma exacerbations are not common [79], while anaphylaxis has been reported anecdotally [85,86], and no fatality has been registered. A very low percentage of patients discontinues SLIT because of side effects [20,39,85]. Contraindications include serious immune-associated diseases (e.g., severe immunodeficiencies), malignancies, chronic and disabling diseases (e.g., major cardiovascular disease, chronic infections, severe psychological disorders) [87]. Beta-blocker treatment is a relative contraindication. If uncontrolled asthma is a risk factor for developing serious adverse events in response to SCIT [69,88], it is reasonable to infer that in these patients SLIT is contraindicated [85]. The daily SLIT dose should temporarily not be given in the following circumstances: bronchospasm, acute febrile illness, oral injury or ulceration (e.g., dentalextraction, aphtae).

## 10. Indications

The impact of SLIT on asthma is often assessed as a secondary outcome in studies on IgE-mediated allergic rhinitis. So, SLIT should be used in children with controlled mild and moderate asthma [16] or controlled severe asthma [69] associated with allergic rhinoconjunctivitis. There is a conditional recommendation on the use of SLIT in children when allergic asthma is isolated because of the moderate or low quality of evidence [16] that does not allow defining a clear recommendation [89]. However, the EAACI Guidelines [2] state that the available evidence support the efficacy of HDM SLIT for pediatric asthma and recommend HDM SLIT drops for children with controlled HDM-driven allergic asthma as an add-on treatment. For other allergens, the prescription clearly depends on the product and the type of the eliciting allergen. SLIT tablets or SLIT drops with documented efficacy should be given to asthmatic children with allergy to grass, birch, or other pollens. Low-quality data support the use of SLIT in children with allergy to *Alternaria* and cat dander [69].

In polysensitized children constituting the majority of those with a pollen allergy, the molecular-based diagnosis would permit the identification of genuine sensitizers and cross-reactive panallergens [90]. The effectiveness of AIT would possibly be increased by prescribing AIT only for genuine allergens. In the pollen—food allergy syndrome [91], SLIT does not improve symptoms to cross-reacting foods. It should be carefully excluded that asthma is elicited by foods [92].

Besides the severity of manifestations, SLIT should be considered if avoidance of the identified relevant inhalant allergens is not effective or is impracticable as the most advantageous treatment set-up. SLIT should be started when pharmacotherapy is protracted, e.g., for more than 3 months, or induces side effects. The cost and the presumed adherence to SLIT are to be considered. During SLIT, children should always receive correct pharmacotherapy. SLIT efficacy should be ascertained by reduction of frequency and severity of symptoms, use of medication, and improvement of lung function. The evaluation of SLIT results should be made following at least six months of pre-coseasonal SLIT for pollen or six to twelve months for perennial allergens and SLIT can be discontinued when patients get worse. There is no absolute age limitation for SLIT administration. Even though the efficacy and safety of SLIT has been shown in children of 3 years of age, evidence is scarce [81,82,93]. Therefore, in preschool children, SLIT should be prescribed after carefully assessing risks and benefits and SLIT drops should be preferred. There are no data suggesting that children receiving SLIT are at a higher risk for the COVID-19 infection. It is recommended to carry on the administration of SLIT during the COVID-19 pandemic. Patients with suspected or confirmed infection with COVID-19 should discontinue the treatment [94]. Finally, in children with rhinoconjunctivitis caused by grass or birch, it has been shown that some SLIT products can be a feasible option not only for controlling symptoms, but also for preventing the onset of asthma [13,95]. A minimum of 3 years course is generally recommended to obtain a preventive effect [93].

## 11. Conclusions

SLIT is a nice example of precision medicine for allergen-driven asthma. There has been a significant progress in SLIT over the last years with introduction of new formulations. Recently approved SLIT products have been investigated in large trials, mainly in adults with asthma or in patients with allergic rhinitis, and there is a need in studies on their use in asthmatic children. Generally, SLIT appears to be safe and effective as an additional treatment in most children with controlled IgE-mediated asthma due to more common allergens. However, products differ in characteristics and efficacy. A distinction of products is necessary to avoid confusion and predict benefits.

## Figures and Tables

**Table 2:** Studies on the long-term effect and preventive role of SLIT in allergic children.

Author, Year	Type of Study	Aim of the Study	Allergen Extract	Patients’ Age (y)	Population (Total Patients, Active/Control Groups)	AIT Duration (Months)	Main Results
Novembre et al., 2004 [73]	RCT	Determine whether SLIT is effective in reducing ocular and nasal symptoms and the development of asthma in allergic children	Grass pollen	5–14	113 (54/59)	4	SLIT ↓ seasonal allergic rhinitis symptoms and ↓ the development of seasonal asthma
Marogna et al., 2008 [74]	RCT	Evaluate the clinical and preventive effects of SLIT in allergic children	Grass pollen	5–17	216 (144/72)	36	SLIT reduced the onset of new sensitizations and mild persistent asthma and ↓ bronchial hyperreactivity
Valovirta et al., 2018 [75]	RDBPC	Investigate the effect of grass SLIT on the risk of developing asthma	Grass pollen	5–12	812 (398/414)	36	SLIT ↓ the risk of experiencing asthma symptoms or using asthma medication ↓ Total IgE, ↓sIgE and SPT for grass pollen

RCT, randomized controlled study, SPT, skin prick test.

**Table 3:** Studies evaluating safety of sublingual immunotherapy in young children.

Author, Year	Allergen Extract	Age (Year)	Population (Total Patients, Active/Control Groups If Applicable)	AIT Duration (Months)	Main Results
Agostinis et al., 2004 [81]	HDM, grass	1–3	36	12–36	SLIT can be safely administered to very young children.
Di Rienzo et al., 2005 [78]	Various	3–5	126	24	SLIT is safe in children under the age of 5 years.
Fiocchi et al., 2005 [82]	Various	3–6	65	12	High-dose immunotherapy in children younger than 5 years is as safe as in older children.
